# Efficient calculation of g-factors for CG-SENSE in high dimensions: noise amplification in random undersampling

**DOI:** 10.1186/1532-429X-16-S1-W28

**Published:** 2014-01-16

**Authors:** Mehmet Akcakaya, Tamer A Basha, Warren J Manning, Reza Nezafat

**Affiliations:** 1 Medicine, Beth Israel Deaconess Medical Center, Harvard Medical School, Boston, Massachusetts, USA; 2 Radiology, Beth Israel Deaconess Medical Center, Harvard Medical School, Boston, Massachusetts, USA

## Background

SENSE [1,2] is one of the most used parallel imaging techniques. In [1], uniform undersampling was employed to efficiently reconstruct an unalised image, whereas in [2], a conjugate gradient-based method (CG-SENSE) was used for reconstruction with arbitrary trajectories. SENSE framework allows the calculation of g-factors, characterizing the noise amplification for a given k-space trajectory and coil configuration [1]. However, calculation of g-factors for arbitrary trajectories in high dimensions is time-consuming [3]. Furthermore, noise characteristics of random undersampling, used in compressed sensing, is not well-understood. In this work, we use a Monte-Carlo (MC) method for fast calculation of g-factors for CG-SENSE similar to [4,5] and apply it to random Cartesian undersampling trajectories. **Theory: **SENSE involves a pre-whitening step [1,2], thus without loss of generality, we assume white noise. SENSE reconstruction solves min_m _||**y - Em**||_2_, where **E **is the system matrix, and **y **are the undersampled measurements. The g-factor for the k^th ^voxel is given by g_k _= √([E*E]^-1^_k,k _[E*E]_k,k_). Inverting E*E is not feasible in high dimensions. Instead we note the g_k _corresponds to the k^th ^diagonal of the reconstruction noise covariance matrix (for normalized coil sensitivities), where **n**_recon _= (E*E)^-1^E*n_meas_, and **n**_meas _is measurement noise with identity covariance matrix. We calculate the sample correlation matrix using a MC approach (since sample mean goes to 0), as 1/(p-1)∑_p _**n**^p^_recon _(**n**^p^_recon_)* for p instances of **n**_recon_. Note we only calculate and store the diagonal elements of this matrix, significantly increasing efficiency.

## Methods

The MC method was first verified in a numerical simulation, where the g-factor was explicitly calculated for a 2D coil configuration, to determine how many MC simulations suffice. Whole-heart imaging was performed with an isotropic resolution of 1.3 mm using a 32-channel coil array. Two 4-fold accelerated acquisitions were performed, one with uniform undersampling (2 × 2 in the k_y_-k_z _plane) and one with random undersampling. Coil sensitivity maps were exported. Images were reconstructed using SENSE (for uniform) and CG-SENSE (for both). g-factors were also calculated with the proposed approach.

## Results

**Figure 1:**
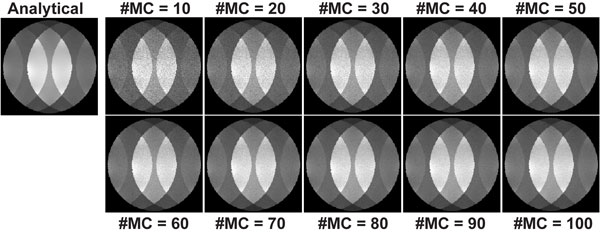
**g-factor maps calculated from numerical simulations using point-by-point analytical evaluation, as well as the described Monte-Carlo method for various number of simulations (#MC)**. The Monte-Carlo based approach converges after ~50 simulations.

**Figure 2:**
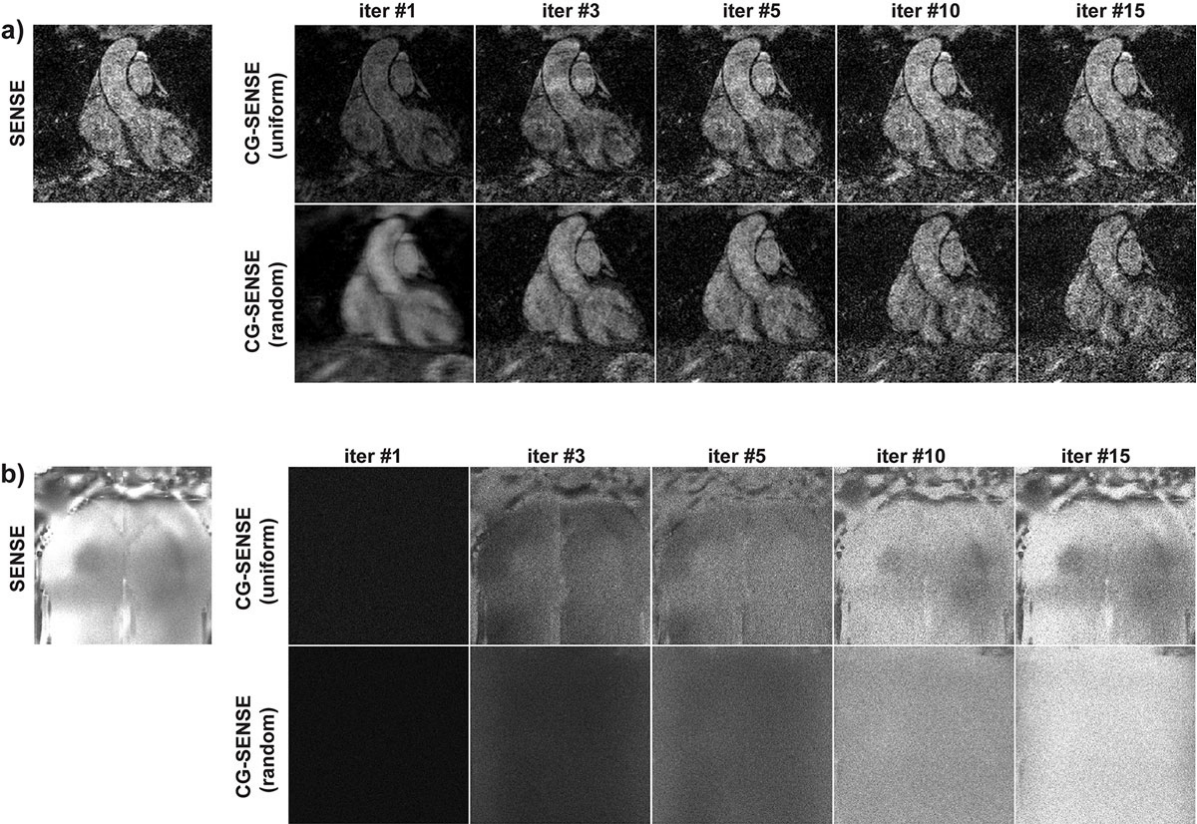
**(a) Reconstructions from two 4-fold accelerated acquisitions with uniform and random sampling (zoomed into the heart)**. CG-SENSE with uniform undersampling converges in 10-15 iterations, whereas CG-SENSE with random undersampling converges in ~5 iterations (also exhibiting the semi-convergence property attributed to CG-SENSE). (b) The corresponding g-factor maps from 50 MC simulations (depicting the whole slice). g-factor maps for uniform undersampling with CG-SENSE converges to the SENSE maps in 10-15 iterations, exhibiting the folding patterns associated with SENSE reconstructions. g-factor maps for random undersampling are more homogenous, amenable to denoising with a fixed threshold (semi-convergence is also exhibited in these maps). g-factor values taken near the ascending aorta are 1.80 for SENSE; 0.55,1.26,1.60,1.80,1.79 for CG-SENSE with uniform undersampling (iterations 1,3,5,10,15 respectively); and 0.46,0.76,1.09,1.85,2.45 for CG-SENSE with random undersampling (iterations 1,3,5,10,15 respectively).

Figure 1 shows the results of numerical simulations, indicating the method converges in ~50 MC simulations. Figure 2 shows the reconstructions associated with the two undersampling patterns and reconstructions, and the corresponding g-factors respectively. The results exhibit the semi-convergence property for random undersampling but not for uniform. Furthermore, the g-factor for random undersampling is smaller at its convergent point than for uniform.

## Conclusions

g-factors for random undersampling is better than those for uniform at high k-space dimensions and high acceleration rates.

## Funding

NIH:K99HL111410-01; R01EB008743-01A2.

## References

[B1] Pruessmann MRM 1999

[B2] Pruessmann MRM 2001

[B3] Liu ISMRM 2008

[B4] Thunberg MRI 2007

[B5] Robson MRM 2008

